# Reduction of elective lymph node volume in radiotherapy of early anal squamous cell cancer: a comparative study between two Swedish university hospitals

**DOI:** 10.2340/1651-226X.2024.20336

**Published:** 2024-04-08

**Authors:** Sofia Heyman, Mats Perman, Calin Radu

**Affiliations:** a Department of Oncology, Institute of Clinical Sciences, Sahlgrenska Academy, University of Gothenburg, Gothenburg, Sweden; b Department of Oncology, Sahlgrenska University Hospital, Region Västra Götaland, Göteborg, Sweden; c Department of Immunology, Genetics and Pathology, Uppsala University, Uppsala, Sweden

**Keywords:** Anal cell carcinoma, chemoradiotherapy, pelvic irradiation, regional recurrence, external iliac lymph nodes, presacral lymph nodes

## Abstract

**Background:**

Anal squamous cell cancer (ASCC) in early stages (T1–2N0M0) is treated with chemoradiotherapy with a 3-year overall survival (OS) exceeding 90%. In Swedish guidelines, it has been optional to include the external iliac and presacral lymph node (LN) stations in radiotherapy (RT) treatment fields in early ASCC. Two Swedish hospitals treating ASCC (SU: Sahlgrenska University Hospital; UU: Uppsala University Hospital) have chosen different approaches since 2010.

**Material and methods:**

This study included consecutive patients with early ASCC (T1–2N0M0) treated between 2010 and 2017 at both sites (SU *n* = 70; UU *n* = 46). Data were retrieved from medical records and RT charts.

**Results:**

At SU, the external iliac and presacral LN stations were included in elective LN irradiation in 96.8% (*n* = 60) and 95.2% (*n* = 59) patients compared to 2.4% (*n* = 1) and 29.3% (*n* = 12) at UU. The mean elective LN volume was 2,313 cc (interquartile range [IQR] 1,951–2,627) in the SU cohort compared to 1,317 cc (IQR 1,192–1,528) in the UU cohort, *p* < 0.0001. No case of regional LN recurrence was seen in either cohort. Disease specific survival (DSS) at 5 years was 95.7% (confidence interval [CI] 90.1–100.0) in the SU cohort and 97.8% (CI 93.2–100.0) in the UU cohort (p 0.55). OS at 5 years was 84.5% (CI 76.1–93.0) in the SU cohort and 82.6% (CI 69.6–89.1) in the UU cohort (p 0.8).

**Interpretation:**

We found no differences in regional recurrence, DSS or OS between the cohorts treated with different elective LN volumes. In this population-based study, reduction of RT volume in early ASCC did not lead to inferior outcome.

## Introduction

Anal squamous cell cancer (ASCC) in early stages (T1–2N0M0, UICC TNM7) is treated with radiotherapy (RT) with addition of chemotherapy in most cases (chemoradiotherapy; CRT). Recent cohort studies indicate a 3-year disease free survival (DFS) of 84–85% and an overall survival (OS) of 92% in early ASCC treated with RT alone or CRT [1, 2]. The risk of local recurrence in early ASCC treated with CRT is low and has been reported in 5.4% of the patients [2]. If left untreated, the inguinal lymph nodes (LN), are the most common site of regional recurrence in these patients [3, 4].

In Sweden, ‘Australasian Gastrointestinal Trials Group’ (AGITG) guidelines have been widely used in RT planning [5]. According to these guidelines all major pelvic LN stations should be covered for all stages of ASCC. However, the AGITG guidelines propose that the cranial border can be lowered, and inguinal LN stations be omitted in selected cases of early T1N0 with major comorbidities and low risk of recurrence [5]. In the Swedish guidelines for treatment of ASCC, it has been optional to lower the cranial border and exclude the external iliac and presacral LN stations in early ASCC (T1–2N0). One site treating ASCC, Uppsala University Hospital (UU), has consequently opted for a reduced elective volume in early ASCC excluding the external iliac and presacral LN stations since prior to 2010. At the other sites, including Sahlgrenska University Hospital (SU), the standard practice has been to include all elective LN stations regardless of tumour stage.

Normal tissue toxicity is dependent on both RT dose and volume [6]. Reduction of elective LN dose and volume is therefore of major importance as long as oncologic outcome is not compromised. The aim of this study was to compare two cohorts treated with different elective LN volumes. Our hypothesis was that reduction of elective LN volumes in early ASCC does not impair disease specific survival (DSS) or OS.

## Material and methods

### Study population

**Figure 1:**
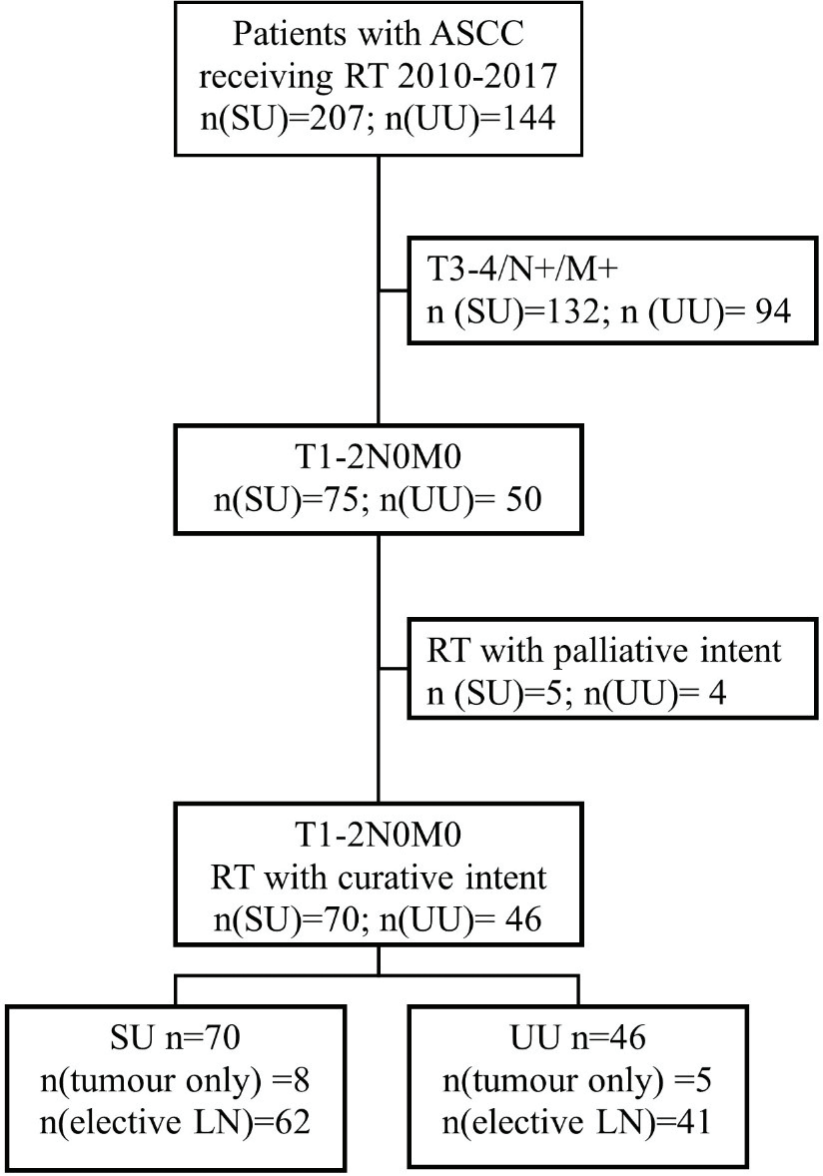
Flowchart. Patients with early ASCC (T1–2N0M0, UICC TNM7) treated with curative intent were included. Patients treated with palliative intent due to serious concurrent disease were excluded. ASCC: anal squamous cell cancer; RT: radiotherapy; SU: Sahlgrenska University Hospital; UU: Uppsala University Hospital; LN: Lymph node.

We identified all patients with ASCC treated with RT/CRT at SU and UU during January 2010 to December 2017. Patients with early tumours defined as T1–2N0M0 (UICC TNM7), treated with a curative intent were included in this study (Figure 1). Data regarding RT dose, volume and technique were retrieved from RT charts. Data regarding tumour stage and characteristics, chemotherapy, oncologic outcome, and survival were retrieved from medical records.

The standard methods used for staging were procto/rectoscopy, digital examination, and pelvic magnetic resonance imaging (MRI). In addition, [18F]-fluorodeoxyglucose positron emission tomography with computed tomography (PET-CT) was used in staging in 87.1% (*n* = 101) of all patients in both cohorts. If PET-CT was not performed, CT of the thorax, abdomen and pelvis was performed to screen for distant metastases. Tumours 50 mm or less on MRI without engagement of adjacent organ were considered as T1–2 (UICC TNM7). LN status was considered negative (N0) if there were no signs of LN metastases on MRI, and on MRI and PET-CT when both methods were used. Follow-up after treatment consisted of regular clinical examinations by an oncologist (SU cohort) or a surgeon (UU cohort). At SU, treatment response was evaluated with PET-CT and digital examination. At UU, treatment response was evaluated with digital examination and imaging only in selected cases.

**Figure 2:**
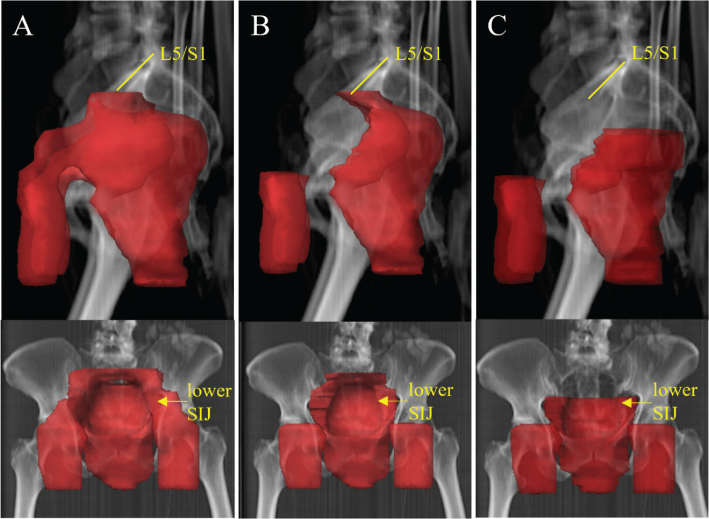
Differences in delineation of elective LN volumes. This is a real case of early ASCC treated at SU 2010–2017. To the left (A) the clinical target volume (CTV) from the original RT plan is seen. CTV includes the inguinal, internal/external iliac, mesorectal, presacral LN stations up to the border between the lumbar spine and sacrum, i.e. the promontorium. This represents the common target delineation in the SU cohort. In the middle (B) the external iliac LN stations have been excluded. To the right (C), the presacral, external and upper internal iliac LN stations have been excluded. The cranial border of CTV is at the caudal border of the sacroiliac joints. This represents the common target delineation in the UU cohort. LN: Lymph node; ASCC: anal squamous cell cancer; SU: Sahlgrenska University Hospital; RT: radiotherapy; UU: Uppsala University Hospital. L5/S1: Fifth lumbar vertebrae/First sacral vertebrae; SIJ: sacroiliac joint.

The RT technique used was either three dimensional conformal RT (3DCRT) with sequential boost or intensity-modulated radiotherapy (IMRT)/volumetric modulated arc therapy (VMAT) with simultaneously integrated boost (SIB). The dose/fraction, number of fractions, final dose to tumour and elective LN volume was registered. Equivalent dose in 2 Gray (Gy) fractions (EQD2) with alfa/beta 10 was calculated to compare fractionation schemes. The delineation of target volume (Clinical target volume; CTV) was evaluated in each case to determine which LN stations that had been included. The planned target volume (PTV) of the nodal stations was used as a measure of the total elective LN volume. Moreover, we determined which LN stations that were included in the prescribed and delineated treatment volumes. LN volumes were categorised according to the AGITG guidelines [5]. Mesorectum was defined as mesorectum up to the border of the lower sacroiliac joints. The presacral space was defined as the space in front of the sacrum with a 2 cm margin to the bone. We made a distinction between upper internal iliac LN station and lower internal iliac LN station localised below the sacroiliac joints (Figure 2). A 6-8 mm margin was used from CTV to PTV at both SU and UU.

We recorded overall treatment time (OTT), interruptions in RT, and concomitant chemotherapy in terms of content, dosing, interval, and interruptions.

DFS was defined as time from treatment (date of last RT fraction) to disease recurrence. Disease recurrence was defined as persistent tumour growth, recurrent tumour growth, regional LN recurrence or distant metastases. DSS was defined as time from treatment to death due to anal cancer. OS was defined as time from treatment to death, regardless of disease recurrence. Follow-up was complete for all patients up to 5 years or death.

### Statistical analysis

Categorical variables were described as frequencies or percentages with 95% confidence interval (CI), and were compared using the chi-square test. Survival analysis was performed using the Kaplan-Meier method, and differences in survival were calculated with log-rank test. Quantitative data were presented as mean or median values with range, CI (95%) or interquartile range (IQR). Quantitative data were compared using unpaired two-sided *t*-test. *P*-values <0.05 were considered statistically significant. Statistical Package for the Social Sciences (SPSS) version 29 (SPSS Inc., Chicago, Illinois, USA) was used for statistical analysis.

### Ethical approval

The study was conducted with respect to the ‘General Data Protection Regulation’ (GDPR), and was ethically approved by the Swedish Ethical Review Authority (registration number 2021–01735).

## Results

### Treatment

The groups were similar with respect to age, sex, and tumour stage (Table 1). The median tumour size was significantly larger in the UU cohort (*p* < 0.05). Testing for p16, as a surrogate marker of human papilloma virus (HPV) association, was introduced in routine work-up around 2015. It was performed in only 38.8% of all patients (*n* = 45) and p16 was positive in 91.1% (*n* = 41) of the tested cases. A subgroup (*n* = 13; 11.2%) underwent primary local excision of ASCC followed by postoperative RT/CRT, whereas the majority (*n* = 103; 88.8%) received RT/CRT as their primary treatment.

**Table 1:** Baseline patient and tumour characteristics.

Patient characteristics	SU (*n* = 70)				UU (*n* = 46)				*P*
		Range	*n*	%		Range	*n*	%	
Age, md (y)	68.7	36–93			65.1	45–90			0.38
Age ≥ 75 years (*n*; %)			17	24.3			11	23.9	0.96
Female (*n*; %)			51	72.9			30	65.2	0.42
Male (*n*; %)			19	27.1			16	34.8	0.42
Pelvic MRI (*n*; %)			70	100			46	100	na
PET-CT (*n*; %)			59	84.3			42	91.3	0.27
T1 (*n*; %)			22	31.4			11	23.9	0.23
T2 (*n*; %)			48	68.6			35	76.1	0.23
Tumour size, md (mm)	21	7–49			30	6–48			< 0.05

SU: Sahlgrenska University Hospital; UU: Uppsala University Hospital; md: median; y: years; T1: tumour stage 1; T2: tumour stage 2; MRI: magnetic resonance imaging; PET-CT: [18F]-fluorodeoxyglucose positron emission tomography with computed tomography.

Chemotherapy was more frequently used in the UU cohort. At SU, 67.1% (*n* = 47) of the patients received chemotherapy compared to 84.8% (*n* = 39) of the UU patients. The use of concomitant chemotherapy got more common over time in both cohorts (Table 2). The two most used regimens were mitomycin C (MMC; 10 mg/m^2^) in combination with either capecitabine (Cape; 825 mg/m^2^ BID) or 5-fluoruracil infusion (5FU; 4,000 mg/m^2^ 4-day infusion) (Table 2).

**Table 2:** Details of treatment and treatment failures.

Treatment	SU (*n* = 70)				UU (*n* = 46)				*P*
		Range	*n*	%		Range	*n*	%	
**Chemotherapy**									
Patients receiving chemotherapy			47	67.1			39	84.8	0.05
2010–2013			13 of 30	43.3			11 of 16	68.8	0.01
2014–2017			4 of 40	85.0			28 of 30	93.3	0.89
Chemotherapy regimen									
Cape-MMC			0	0			7	17.9	
5FU-MMC			46	97.9			32	82.1	
5FU-Cis			1	2.1			0	0	
**Radiotherapy**									
IMRT/VMAT (*n*; %)			58	82.9			46	100	< 0.05
Dose to primary tumour, md (Gy)									
Physical dose	54.0	54–60			50.8	36–64			< 0.01
Dose per fraction	2.0	2–2			2.15	2–2.23			
EQD2	54.0	54–60			51.7	36–64			< 0.01
Dose to elective LN, md (Gy)									
Physical dose	46	40.7–48.6			41.4	40.7–48.6			< 0.01
Dose per fraction	1.8	1.5–2			1.8	1.5–2			
EQD2	44.0	36–46.4			40.7	40–47.9			< 0.01
OTT, md (days)	40	23–57			32	25–42			
**Surgery**									
Local excision prior to RT			6	8.6			7	15.2	0.40
Salvage surgery after RT			4	5.7			6	13.0	0.44
**Treatment failure**									
Recurrence (*n*; %)			7	10			7	15.2	0.55
Persistent tumour only			2	2.9			5	10.9	
Local recurrence only			2	2.9			2	4.3	
Regional LN recurrence			0	0.0			0	0.0	
Distant metastases			3	4.3			0	0.0	

SU: Sahlgrenska University Hospital; UU: Uppsala University; Cape-MMC: capecitabine with mitomycin; 5FU-MMC: 5-fluorouracil with mitomycin; 5FU-Cis: 5-fluorouracil with Cisplatin; IMRT/VMAT: intensity modulated radiotherapy/volumetric modulated arc therapy; md: median; Gy: Gray; EQD2: Equivalent dose in 2 Gy fractions; LN: lymph node; OTT: overall treatment time; RT: radiotherapy.

In the SU cohort, 82.9% (*n* = 58) received IMRT/VMAT with SIB compared to 100% (*n* = 46) in the UU cohort (Table 2). Eight SU patients (11.4%) and five UU patients (10.9%) received RT with curative intent to primary tumour only. Elective LN irradiation was given in a similar extent in both cohorts, 88.6% (*n* = 62; SU) vs 89.1% (*n* = 41; UU).

**Figure 3:**
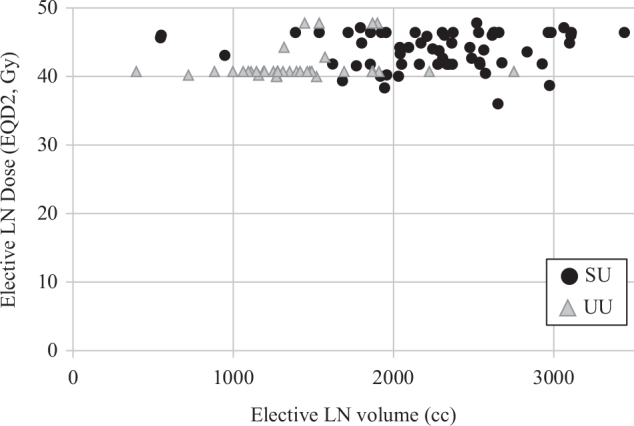
Elective LN dose (EQD2, Gy) versus elective LN volume (cc) in patients with early ASCC treated at SU (black dots) and UU (grey triangles) with curative intent during 2010–2017. The elective LN volume equals the planned target volume (PTV) on the RT plan. The UU patients are clustered in the upper left corner whereas the SU patients are clustered in upper the right corner. LN: Lymph node; EQD2: Equivalent dose in 2 Gy fractions; Gy: Gray; cc: cubic centimetres; ASCC: Anal squamous cell cancer; SU: Sahlgrenska University Hospital; UU: Uppsala University Hospital.

There was a variation in and between the cohorts in both RT dose and target volume (Figure 3). The median dose (EQD2) to site of primary tumour was 54.0 Gy (range 54–60) in the SU group and 51.7 Gy (range 36–64) in the UU group. In patients receiving elective LN irradiation, the median dose (EQD2) to elective LN was 44.0 Gy (range 36–46.4) in the SU group and 41.4 Gy (range 40.0–47.9) in the UU group (Table 2).

Median delineated elective LN volumes were significantly larger in the SU group, 2,313 cc (IQR 1,951–2,627) compared with the UU group, 1,317 cc (IQR 1,192–1,528), *p* < 0.001. There were no differences in body size assessed by body mass index (BMI) or body surface that could explain the differences in elective treatment volume (data not shown). The external iliac LN station was included in delineated elective LN volume in only one UU patient (*n* = 1; 2.4%) but was consistently included in the SU group (*n* = 60; 96.8%). The presacral and upper internal iliac LN stations were included in 95.2% (*n* = 59) of the SU patients compared with 29.3% (*n* = 12) of the UU patients (Table 3). UU patients prescribed RT to this compartment had with only one exception anal tumours extending into the rectum. In the SU cohort, 95.2% (*n* = 59) of the patients were prescribed elective LN irradiation comprising all available elective LN stations (inguinal, internal/external iliac, mesorectal, presacral, obturator, ischiorectal LN). Only one UU patient (2.4%) had RT including all elective LN stations (Table 3).

**Table 3:** Lymph node stations included in the elective LN volume in patients receiving elective LN irradiation for early ASCC at SU and UU, respectively.

Elective LN volumes	SU (*n* = 62)				UU (*n* = 41)				*P*
	*n*	%	cc	IQR	*n*	%	cc	IQR	
Inguinal regions	62	100.0			40	97.5			0.78
Upper internal iliac vessels	61	98.4			12	29.3			< 0.001
Lower internal iliac vessels	61	98.4			39	95.1			0.89
Obturator space	61	98.4			39	95.1			0.89
Mesorectum	61	98.4			39	95.1			0.89
Ischiorectal fossa	62	100.0			27	65.9			< 0.001
Presacral space	59	95.2			12	29.3			< 0.001
External iliac vessels	60	96.8			1	2.4			< 0.001
LN volume, md (cc)			2,313	1,951–2,627			1,317	1,192–1,528	< 0.0001

The LN volume measured in cc is equal to the planned target volume (PTV) in the RT dose plan.

LN: lymph node; ASCC: Anal squamous cell cancer; SU: Sahlgrenska University; UU: Uppsala University; md: median; cc: cubic centimetres; IQR: Interquartile range.

### Outcome

There were no regional LN recurrences in either cohort. In the SU cohort, 4 patients (5.7%) were diagnosed with either persistent primary tumour (*n* = 2) or recurrent primary tumour (*n* = 2). In the UU cohort, 7 patients (15.2%) were diagnosed with either persistent primary tumour (*n* = 5) or recurrent primary tumour (*n* = 2). Distant spread was seen in 3 patients (4.3%) in the SU cohort but in none of the UU patients (Table 2). All but one patient with locally recurrent or persistent ASCC underwent salvage surgery (*n* = 10; 90.9%). Of these, only one patient had a new recurrence after surgery.

**Figure 4:**
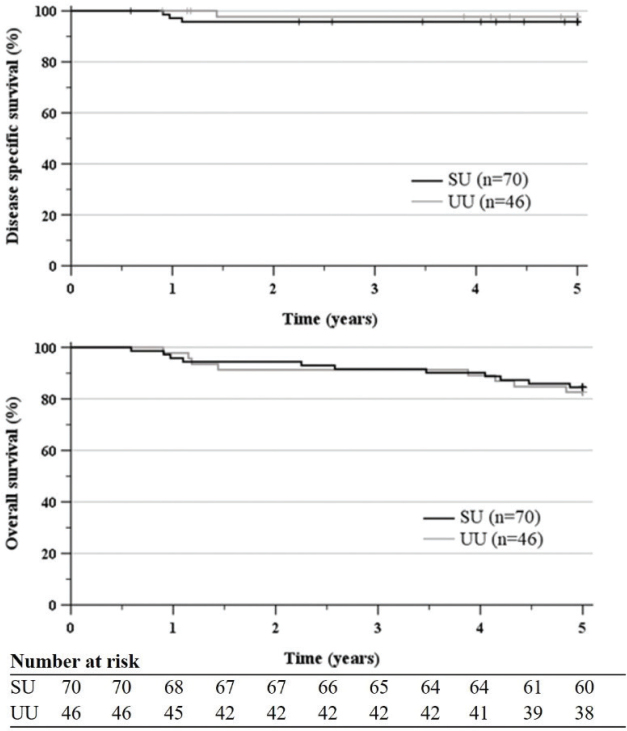
DSS and OS after curative RT/CRT of early ASCC treated 2010–2017 at SU and UU. DSS at 5 years was 95.7% (CI 90.1–100.0) in the SU cohort and 97.8% (CI 93.2–100.0) in the UU cohort. OS at 5 years was 84.5% (CI 76.1–93.0) in the SU cohort and in 82.6% (CI 69.6–89.1) the UU cohort. DSS: disease specific survival; OS: overall survival; RT: radiotherapy; CRT: chemoradiotherapy; ASCC: anal squamous cell cancer; SU: Sahlgrenska University Hospital; UU, Uppsala University Hospital; CI: confidence interval.

DFS at 5 years was 90.0% in the SU cohort (CI 82.6–96.7) and 84.8% in the UU cohort (CI 73.7–94.0), p 0.44. Anal cancer was assessed to be the terminal cause of death in three SU patients (4.3%) and one UU patient (2.2%), respectively. DFS at 5 years was 95.7% (CI 90.1–100) in the SU cohort and 97.8% (CI 93.2–100) in the UU cohort, p 0.55. OS at 5 years was 84.5% (CI 76.1–93.0) in the SU cohort and 82.6% (CI 69.6–89.1) in the UU cohort, p 0.80 (Figure 4).

## Discussion

In this study, we found no differences in regional recurrence, DSS or OS between two population-based cohorts with early ASCC treated with different elective LN volumes. There was a difference in DFS between the cohorts although not statistically significant. The completeness of data, the 5-year follow-up, the similar distribution of other factors than LN volume, all strengthen the validity of our results. The aim of this investigation did not include toxicity assessment. However, we expect significant clinical benefit when decreasing the treatment volume, given the well-established relationship between irradiated volume and long-term toxicity [6, 7].

In the UU cohort, the median tumour size was larger and the RT dose to primary tumour was lower. This may explain the higher rate of tumours deemed as persistent (*n* = 5; 10.9%). Another factor might be differences in treatment evaluation. In the UU cohort, treatment evaluation was performed by a surgeon and in the SU cohort by an oncologist. In our opinion, this might have led to a more active approach to perform early surgery. Unfortunately, pathology reports were not available for analysis of complete pathological response, an indicator of ‘unnecessary’ early salvage surgery. The survival was excellent after salvage surgery in both cohorts.

The study cohorts differed in use of concomitant chemotherapy. The second part of the SU cohort (2014–2017, *n* = 40) received concomitant chemotherapy in a similar extent as the UU cohort (*n* = 46). When we did a post hoc sub analysis comparing the second SU cohort with the UU cohort, the results did not change. This supports our notion that suboptimal chemotherapy at SU did not mask inferior RT volume used at UU.

The inguinal LN station is the most common site of LN metastases both at diagnosis and recurrence of ASCC [4, 8, 9]. Retrospective data indicate that omitting the inguinal LN volume leads to a higher rate of regional LN recurrence in early ASCC [8, 10]. In contrast, isolated metastases in the external iliac and presacral LN stations are rarely seen at diagnosis or recurrence [4, 9]. Moreover, two Norwegian studies reported no regional recurrences in patients with early ASCC treated with reduced volumes terminating at the lower end of the sacroiliac joint [3, 11]. This supports our notion that external iliac and presacral LNs can be omitted in node negative ASCC. The potential benefits of excluding the external iliac and presacral LN stations are obvious given their proximity to both bladder and bowel.

Several studies with a retrospective design have been made comparing outcome and RT with different volumes in ASCC. In the study from Das et al., 5 out of 66 patients treated with reduced RT fields terminating at the lower sacroiliac experienced pelvic regional recurrence, whereas no case of regional recurrence was seen in 89 patients treated with RT fields terminating at the L5/S1 interspace [12]. The study included a mixed patient population and details of disease characteristics in the patients with relapse are not reported. Moreover, the patients were treated between1994 and 2004 and there are differences compared to what is regarded as standard-of-care today. For instance, the patients were not staged with MRI or PET-CT, and RT was delivered with static fields. The DFS (55%) and OS (69%) at 5 years is much lower compared with what is reported from more recent studies [1, 2, 13]. A similar, but smaller Australian study from 2018 reported the oncologic outcome in 51 patients treated with different RT volumes at a single centre 1994–2007 [14]. No difference in outcome was seen between patients receiving standard RT volumes compared with reduced RT volumes terminating at the lower end of sacroiliac joint. However, the groups were small (27 and 24 patients) and comprised mixed tumour stages. Half of the patients were male which indicate either a selected patient population or a different demography of anal cancer in Australia compared to Northern Europe.

A randomised trial with a non-inferiority design would be ideal to address if reduction of treatment volume is safe. ASCC is a rare disease with a good prognosis. The anticipated difference in OS would be small between the groups and it would require a large number of patients to get enough statistical power to answer the question. In the recently published Nordic ASCC RT guidelines, risk-adapted RT strategies are mainly based on studies of patterns of recurrence in anal cancer and other pelvic malignancies. In early ASCC (T1–2N0 not extending into the rectum) omittance of external iliac, upper iliac internal presacral LN stations is a recommended alternative to standard RT according to these guidelines [15]. Our data, which also includes outcome, support this recommendation.

## Conclusions

In this population-based study, reduction of elective LN volumes in RT of early ASCC led to a significant reduction in total treatment volume, without apparent signs of inferior oncological outcome.

## Conflict of interest

The authors report there are no competing interests to declare.

## Data availability statement

The data that support the findings of this study are available on request from the corresponding author [SH]. The data are not publicly available due to ethical restrictions protecting the privacy of the research participants.
